# Secondary Philadelphia Chromosome–Negative B-Cell Acute Lymphoblastic Leukemia Following Prolonged Lenalidomide Maintenance in Multiple Myeloma

**DOI:** 10.14740/jmc5274

**Published:** 2026-03-27

**Authors:** Jessica Thomas, Yagnapriya Ammakola, Waqqas Tai, Afoma Anyadibe, Anuoluwa Oyetoran, Sarang Khan, Ishmael Jaiyesimi

**Affiliations:** a Hematology/Oncology Fellowship Program, Corewell Health William Beaumont University Hospital, Royal Oak, MI, USA; b Internal Medicine Residency, Corewell Health William Beaumont University Hospital, Royal Oak, MI, USA; c Pathology Residency, Corewell Health William Beaumont University Hospital, Royal Oak, MI, USA; d Hematology/Oncology Fellowship Program and Cellular Therapy Program, Corewell Health William Beaumont University Hospital, Royal Oak, MI, USA

**Keywords:** Multiple myeloma, Lenalidomide, B-cell acute lymphoblastic leukemia, Second primary malignancy, Therapy-related leukemia, Maintenance therapy, Inotuzumab ozogamicin, Allogeneic transplantation

## Abstract

Lenalidomide maintenance significantly improves survival in multiple myeloma (MM) but increases the risk of second primary malignancies (SPMs), with the most common hematologic SPMs being myelodysplastic syndromes (MDS) and acute myeloid leukemia (AML). Secondary B-cell acute lymphoblastic leukemia (B-ALL) is rare. We describe a 62-year-old woman diagnosed with immunoglobulin G lambda MM with extramedullary disease. She achieved remission following induction with lenalidomide, carfilzomib, and dexamethasone, followed by autologous stem cell transplantation. Lenalidomide maintenance was continued for over 4 years, then discontinued due to fatigue. Nearly 1 year later, she presented with bruising and thrombocytopenia. Bone marrow biopsy confirmed Philadelphia chromosome–negative B-ALL with a complex karyotype. She achieved remission with mini–hyper-CVD (cyclophosphamide, vincristine, dexamethasone) plus inotuzumab ozogamicin and rituximab, followed by allogeneic transplantation. This case illustrates lenalidomide-associated B-ALL as a rare late complication of maintenance therapy in MM, underscoring the need for vigilance and early diagnostic evaluation.

## Introduction

Multiple myeloma (MM) accounts for approximately 10% of all hematologic malignancies, with increasing incidence reported among men over the age of 50, particularly in high-income countries [1]. Lenalidomide, an immunomodulatory agent, has become a cornerstone of treatment in MM, both in frontline induction regimens—often in combination with proteasome inhibitors and corticosteroids—and as maintenance therapy following autologous stem cell transplantation [2].

Exposure to prior chemotherapy or radiotherapy is a well-recognized risk factor for the development of therapy-related secondary malignancies, a distinct subset of treatment-induced neoplasms [3]. Among these, lenalidomide has been increasingly associated with second primary malignancies (SPMs), particularly when used as long-term maintenance. Solid tumors account for approximately 3–6%, while hematologic malignancies—most commonly myelodysplastic syndromes (MDS) and acute myeloid leukemia (AML)—represent 1–3% of cases [4]. The risk appears to be amplified when lenalidomide is combined with alkylating agents such as oral melphalan [5].

In contrast, lenalidomide-associated secondary B-cell acute lymphoblastic leukemia (B-ALL) is rare, with only limited case series and individual case reports. This form of therapy-related leukemia typically presents several years after initial lenalidomide exposure, with a median latency of 4 to 5 years. Unlike *de novo* B-ALL in older adults where BCR::ABL1 fusion is present in approximately 25–30% of cases, lenalidomide-associated B-ALL demonstrates a lower incidence of BCR::ABL1 positivity and instead shows enrichment for high-risk cytogenetic features, including TP53 mutations and low hypodiploidy, both of which are associated with inferior prognosis and chemoresistance [6, 7].

The underlying pathophysiology remains poorly understood, although clonal evolution driven by selective immune pressure and modulation of the DNA damage response has been proposed. Here, we present the case of a 62-year-old female who developed a secondary Philadelphia chromosome–negative B-ALL a little less than 1 year after discontinuation of lenalidomide maintenance therapy, while undergoing surveillance for MM in remission.

## Case Report

The patient had no significant pre-existing comorbidities at the time of initial MM diagnosis. She was initially evaluated for worsening left hip and lower back pain. Imaging revealed destructive lytic lesions involving the left sixth rib, thoracic and lumbar vertebrae (T6, T7, T9, T11, T12, L1), and a large chest wall mass with suspected rib invasion. Additional findings included a 5.2 cm pancreatic tail mass and a 7 mm right upper lobe pulmonary nodule. A biopsy of the sixth rib confirmed a plasma cell neoplasm, with flow cytometry demonstrating a lambda-restricted plasma cell population. A core biopsy of the pancreatic tail also confirmed plasma cell myeloma. Subsequent monoclonal protein analysis showed an immunoglobulin G (IgG) lambda M-protein of 4.1 g/dL and a free lambda light chain of 55.26 mg/dL. Bone marrow biopsy revealed 20–30% involvement by lambda monotypic plasma cells. Cytogenetic studies demonstrated hyperdiploidy of chromosomes 3 and 7.

She was diagnosed with advanced IgG lambda MM with extramedullary involvement and began induction therapy with lenalidomide, carfilzomib, and dexamethasone, along with monthly zoledronic acid. After four cycles on the ENDURANCE trial, she achieved a very good partial response (VGPR), with normalization of free light chains and unmeasurable monoclonal protein [8]. A subsequent positron emission tomography (PET) scan 3 years later showed no definite evidence of fluorodeoxyglucose (FDG)-avid malignancy, with prior lesions resolved. A follow-up bone marrow biopsy showed no residual plasma cell myeloma, with negative immunofixation and normalized free light chain ratio. She proceeded to autologous stem cell transplantation with high-dose melphalan (200 mg/m^2^) conditioning. Following autologous transplantation, the patient was started on maintenance lenalidomide at 10 mg daily (21 days on/7 days off), as dose escalation to the standard 15 mg was not tolerated due to persistent fatigue. Surveillance studies confirmed sustained remission throughout maintenance therapy. After more than 4 years, lenalidomide was discontinued due to progressive fatigue.

**Figure 1:**
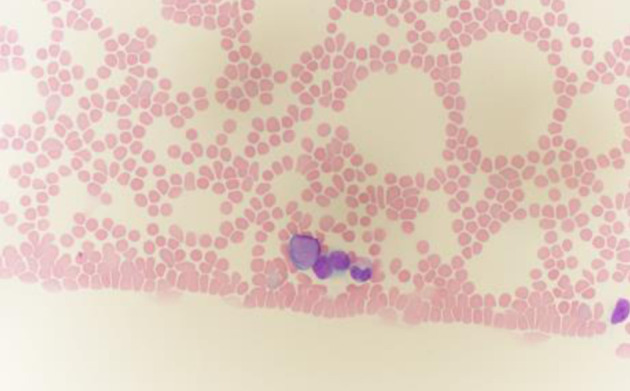
Peripheral blood smear stained with Wright–Giemsa, showing blasts with scant blue cytoplasm (oil immersion, × 1,000). Note the characteristic features of blasts: moderate cell size, high nuclear-to-cytoplasmic ratio, fine chromatin pattern, and inconspicuous nucleoli.

Almost 1 year later, the patient developed sudden-onset bruising, gingival bleeding, and recurrent epistaxis. At her most recent surveillance visit 3 months prior, her platelet count had been normal at 186,000/µL. Repeat laboratory evaluation revealed thrombocytopenia with platelets at 25,000/µL and circulating immature cells. A peripheral blood smear demonstrated a granulocytic left shift with occasional circulating blasts, raising strong suspicion for an underlying hematologic neoplasm (Fig. 1).

**Figure 2:**
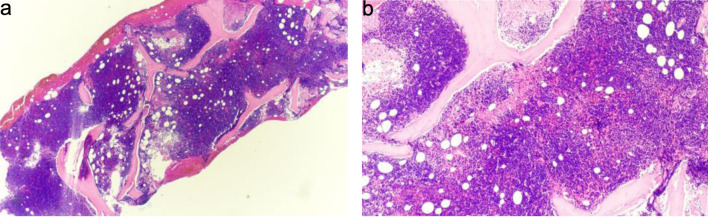
(a) Bone marrow biopsy, hypercellular for age with diffuse replacement by blasts (hematoxylin and eosin stain, × 20). Observe the dense, monotonous cellularity with near-complete effacement of normal hematopoietic elements and marrow architecture. (b) Higher-power view showing sheets of small blue cells consistent with blasts (hematoxylin and eosin stain, × 40). The blasts demonstrate high nuclear-to-cytoplasmic ratio, scant basophilic cytoplasm, finely dispersed chromatin, and inconspicuous nucleoli—features requiring immunophenotyping for definitive lineage assignment. Immunophenotyping by flow cytometry confirmed B-lymphoid lineage (Figs. 4 and 5).

**Figure 3:**
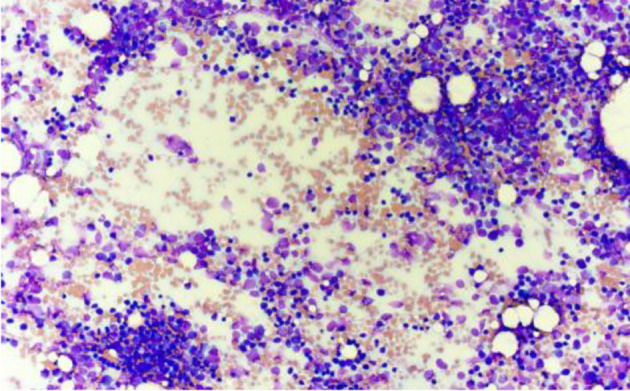
Bone marrow aspirate smear showing increased blast population at low power (Wright–Giemsa stain, × 20). Blasts predominate throughout the aspirate smear with minimal normal hematopoietic precursors visible.

**Figure 4:**
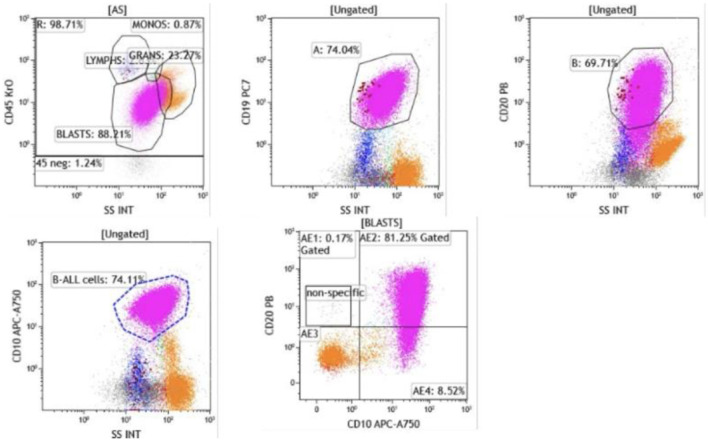
Flow cytometry of bone marrow aspirate. Blasts were identified in the CD45-dim, low side scatter gate, comprising approximately 74% of total events. The blast population expressed CD19, CD10, and CD20 (heterogeneous, moderate to bright), confirming B-lymphoid lineage (CD19, CD10) with early precursor features.

**Figure 5:**
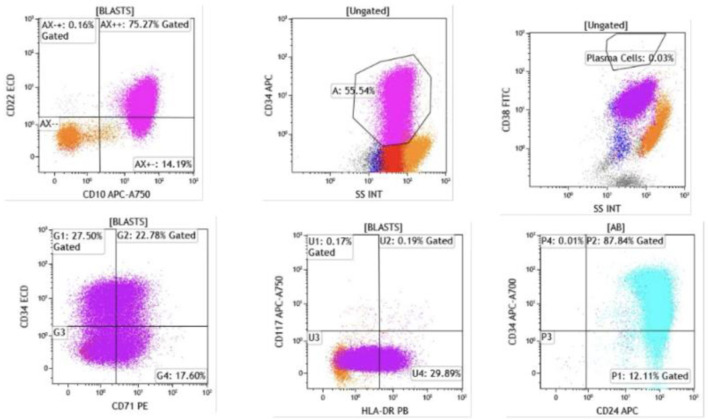
Flow cytometry demonstrated that blasts were positive for CD22 (dim), CD24, CD34 (heterogeneous), CD38, CD45 (dim), CD71, and HLA-DR (heterogeneous). Intracytoplasmic expression of CD79a and terminal deoxynucleotidyl transferase (TdT) was also detected. The presence of TdT and CD34 expression confirms the immature blast phenotype, while CD79a positivity further supports B-cell lineage commitment.

A bone marrow biopsy confirmed the diagnosis of B-ALL (Figs. 2 and 3). Cytogenetic analysis revealed a complex karyotype consistent with poor prognostic features. The ALL fluorescence *in situ* hybridization (FISH) panel demonstrated gain of ABL2 on chromosome 1 (38.6% of nuclei), CDKN2A gene deletion on chromosome 9 (82%), gain of ABL1 on chromosome 9 (34.6%), *MLL* gene rearrangement on chromosome 11 (47.1%), *TCF3* gene involvement on chromosome 19 (60.5%), and *BCR* gene involvement on chromosome 22 (42%), along with loss of chromosome 4 (54%). Although the FISH panel revealed gains of BCR and ABL1 signals individually, there was no evidence of a BCR::ABL1 fusion or t(9;22) translocation, supporting a diagnosis of Philadelphia chromosome–negative (Ph^–^) B-ALL. Concurrent flow cytometry identified a predominant B-lymphoblast population (CD10^+^, CD19^+^, variable CD20, CD22^+^, variable CD34, TdT^+^), confirming the diagnosis (Figs. 4 and 5).

These molecular alterations carry significant prognostic implications. *KMT2A* (*MLL*) gene rearrangements, present in 47.1% of nuclei, are typically associated with aggressive disease and inferior response to standard chemotherapy in adults [7]. The gains of ABL1 and ABL2 without BCR::ABL1 fusion suggest complex genomic instability beyond a simple Philadelphia chromosome–positive disease. *TCF3* gene involvement (60.5%) and *BCR* gene involvement (42%), along with loss of chromosome 4 (54%), further contribute to the high-risk cytogenetic profile [7]. Collectively, these abnormalities define a particularly aggressive subtype of therapy-related B-ALL with increased therapeutic challenges and justify the intensive treatment approach pursued.

The patient was hospitalized for the first cycle of induction chemotherapy following a mini-hyper-CVD regimen combined with inotuzumab ozogamicin and rituximab, along with intrathecal methotrexate and cytarabine for central nervous system (CNS) prophylaxis. This regimen uses an attenuated hyper-CVAD backbone with 50% dose reductions of cyclophosphamide and dexamethasone, omission of anthracycline, 75% dose reduction of intravenous methotrexate, and 83% dose reduction of intravenous cytarabine, combined with low-dose weekly inotuzumab ozogamicin (0.6 mg/m^2^ day 1, 0.3 mg/m^2^ days 8 and 15) and rituximab 375 mg/m^2^ on days 1 and 8 of each cycle. This approach is consistent with standard protocols for Ph^–^ B-ALL in older adults [9]. Cerebrospinal fluid (CSF) cytology and flow cytometry were negative for CNS involvement.

The initial hospital course was complicated by pancytopenia attributable to both disease burden and chemotherapy-induced myelosuppression. She developed an acute subdural hematoma, which was evaluated by neurosurgery and managed conservatively with serial imaging demonstrating stability on repeat CT scans. She experienced an episode of neutropenic fever, which was resolved with antibiotic therapy.

She was subsequently admitted for the second cycle. During this admission, a post-induction bone marrow biopsy performed to assess response after cycle 1 demonstrated complete remission, with concurrent flow cytometry showing no evidence of acute leukemia and cytogenetics reverting to a normal female karyotype. She proceeded with cycle 2, which comprised methotrexate and cytarabine in combination with inotuzumab ozogamicin and rituximab, plus intrathecal methotrexate and cytarabine. CSF studies remained negative for CNS involvement. She tolerated the second cycle without significant complications.

She then received cycle 3, which followed the same regimen as cycle 1 except that inotuzumab ozogamicin was omitted per protocol, as the planned course limited its use to two cycles for patients proceeding to hematopoietic cell transplantation (HCT). Vincristine was withheld during this cycle due to grade 3 peripheral neuropathy. She tolerated the third hospitalization well without acute events.

Given her high-risk disease features (including low hypodiploidy, KMT2A rearrangement, and complex karyotype), secondary disease status, prior autologous transplant, and adverse cytogenetics, she proceeded to allogeneic hematopoietic stem cell transplantation approximately 2–3 weeks after completing cycle 3 [10]. The donor was a 7/8 human leukocyte antigen (HLA)-matched unrelated individual, and transplantation was performed in May 2025. Conditioning consisted of fludarabine 150 mg/m^2^ (30 mg/m^2^ daily for 5 days) and busulfan with pharmacokinetically targeted exposure to an area under the curve (AUC) of 4,000 µmol·min over 2 days. Graft-versus-host disease (GVHD) prophylaxis followed the OPTIMIZE trial protocol with post-transplant cyclophosphamide (25 mg/kg), tacrolimus, and mycophenolate mofetil [10]. Post-transplant bone marrow evaluation approximately 6 weeks later (July 2025) demonstrated complete morphologic remission with no minimal residual disease (MRD) by Clonoseq (a next-generation sequencing-based assay) in both the myeloma and B-ALL clones.

Her post-transplant course was complicated by grade II gastrointestinal GVHD, presenting with refractory nausea and vomiting. Endoscopic biopsies revealed increased apoptotic bodies throughout the upper and lower gastrointestinal tract. She was treated with intravenous corticosteroids, transitioned to an oral taper, and remains clinically controlled on supportive therapy. At approximately 8 months post-transplant, she continues close follow-up with ongoing monitoring for GVHD and routine surveillance for disease relapse.

## Discussion

The development of Ph^–^ B-ALL in this patient—nearly 1 year after discontinuing more than 4 years of lenalidomide maintenance therapy—raises concern for a therapy-related secondary hematologic malignancy. Although most secondary hematologic cancers associated with lenalidomide involve MDS or AML, emerging case reports have described rare instances of lenalidomide-associated B-ALL. The latency period, absence of prior BCR::ABL fusion, complex cytogenetics, and aggressive clinical course in this patient further support the hypothesis of therapy-induced leukemogenesis rather than *de novo* disease. Her case underscores the importance of long-term surveillance for atypical secondary malignancies in patients on extended lenalidomide therapy, particularly those with prior exposure to alkylating agents or autologous stem cell transplant.

While lenalidomide exposure represents a plausible etiologic factor in this case, it is important to acknowledge other potential contributors to therapy-related leukemogenesis. The patient’s prior exposure to high-dose melphalan conditioning before autologous stem cell transplantation may have independently increased her risk of secondary malignancies. Alkylating agents, particularly melphalan, are well-established contributors to therapy-related myeloid neoplasms and may also predispose to lymphoid malignancies. Additionally, the cumulative effect of multiple lines of cytotoxic therapy, including proteasome inhibitors and corticosteroids, could have contributed to genomic instability. The close temporal relationship between lenalidomide discontinuation and B-ALL development, combined with existing literature on lenalidomide-associated lymphoid malignancies, supports lenalidomide as a significant contributing factor; however, a multifactorial etiology involving prior alkylating agent exposure cannot be excluded.

With improvements in overall survival and progression-free survival (PFS) for MM, the incidence of SPMs has become increasingly recognized. The pathogenesis of SPMs is multifactorial, influenced by intrinsic factors such as age, race, sex, genetic predisposition, and comorbidities, as well as extrinsic factors including smoking and sun exposure [11]. Lenalidomide, an immunomodulatory agent, combined with dexamethasone has become a mainstay of treatment for MM across age groups. The randomized phase III SWOG S0777 trial demonstrated a PFS benefit of 41 months with a hazard ratio (HR) of 0.742 (0.594–0.928), and a median overall survival not reached [12]. Similarly, in the phase III Myeloma XI trial, lenalidomide maintenance significantly improved PFS compared to placebo in newly diagnosed patients, with an HR of 0.45 [13].

A meta-analysis of nine trials including 3,254 patients found that the 5-year cumulative incidence of all SPMs was significantly higher in patients who received lenalidomide compared to those who did not: 6.9% (95% confidence interval (CI) 5.3–8.5) versus 4.8% (95% CI 2.0–7.6), with an HR of 1.55 (95% CI 1.03–2.34; P = 0.037). When stratified by malignancy type, solid SPMs showed similar incidence between groups—3.8% (95% CI 2.7–4.9) with lenalidomide versus 3.4% (95% CI 1.6–5.2) without (HR 1.1, 95% CI 0.62–2.00; P = 0.72). However, hematologic SPMs demonstrated a marked increase: 3.1% (95% CI 1.9–4.3) with lenalidomide versus 1.4% (95% CI 0.0–3.6) without, yielding an HR of 3.8 (95% CI 1.15–12.62; P = 0.029). While lenalidomide contributes to SPM risk, high-dose melphalan conditioning for autologous stem cell transplantation also plays a significant role in therapy-related hematologic malignancies, making it difficult to isolate the independent contribution of maintenance lenalidomide. MDS and AML are the most commonly reported hematologic SPMs in this setting [4]. A retrospective study identified 32 cases of B-ALL associated with lenalidomide therapy, with a median latency of 4 to 5 years after lenalidomide exposure and nearly 5.5 years after MM diagnosis, similar to our patient’s timeline [14, 15]. Although absolute incidence rates vary across studies, recent analyses suggest lenalidomide-associated B-ALL may occur in approximately 1% of patients on prolonged maintenance therapy.

The rarity of B-ALL as a secondary malignancy in MM patients highlights the need to better understand its underlying mechanisms [14]. Lenalidomide binds to cereblon, a component of the E3 ubiquitin ligase complex involved in DNA repair. This interaction leads to degradation of transcription factors such as IKZF1 and IKZF3, which are essential for B-cell development and may promote clonal B-cell expansion [16]. Immunomodulatory drugs further alter the tumor microenvironment by impairing T-cell and natural killer (NK)-cell function, reducing cytokine signaling (e.g., interleukin-6 (IL-6), tumor necrosis factor-α (TNF-α)), and inhibiting osteoclast activity—mechanisms which may predispose patients to hematologic rather than solid malignancies [17]. Additional contributing factors include autologous hematopoietic cell transplantation (auto-HCT) and prolonged melphalan use [11, 17]. Together, these exposures can promote clonal evolution and complex cytogenetic alterations seen in secondary B-ALL [7].

Secondary B-ALL more frequently harbors TP53 mutations, hypodiploidy, and monosomy 7 or 7q deletions compared to *de novo* B-ALL [6]. In selected cases, lenalidomide discontinuation may lead to regression of the B-ALL clone, as demonstrated in a retrospective study reporting clonal regression in five patients following drug withdrawal, though complete remission was achieved in only a subset [6]. The hyper-CVAD (hyperfractionated cyclophosphamide, vincristine, doxorubicin, and dexamethasone) ± rituximab regimen has shown complete responses in 56% of patients, with an additional 28% achieving incomplete hematologic recovery and 32% reaching MRD negativity. Allogeneic hematopoietic cell transplantation (allo-HCT) remains a potentially curative option, especially in MRD-negative patients [18].

Targeted agents such as inotuzumab ozogamicin (anti-CD22 antibody–drug conjugate) and blinatumomab (a CD19-targeting bispecific T-cell engager) are approved for relapsed or refractory B-ALL [9]. In eligible adults with relapsed or refractory B-ALL, chimeric antigen receptor (CAR) T-cell therapy with brexucabtagene autoleucel or obecabtagene autoleucel may serve as a bridge to allogeneic transplant or, in select cases, as definitive therapy [19, 20]. In medically fit patients with lenalidomide-associated B-ALL, established regimens (e.g., mini–hyper-CVD), novel antigen-targeted therapies, and allo-HCT may provide durable responses. Our patient was treated with mini–hyper-CVD and inotuzumab ozogamicin per published protocols for Ph^–^ B-ALL [9].

Next-generation sequencing (NGS) plays a growing role in defining the molecular landscape of secondary B-ALL. The American Society of Clinical Oncology (ASCO) endorses the use of NGS for both diagnostic classification and MRD monitoring in B-ALL subsets, including secondary disease [21, 22]. NGS was not available in this case, as reflex testing was not authorized for ALL at our center, even though it is routinely applied for other hematologic malignancies. Other case reports, however, have described NGS findings in lenalidomide-associated B-ALL, underscoring its potential role in characterizing disease biology.

### Learning points

This case highlights several important clinical considerations for patients receiving prolonged lenalidomide maintenance. The abrupt onset of severe thrombocytopenia nearly 1 year after discontinuing lenalidomide underscores the need for continued surveillance even after cessation of therapy, with a low threshold for investigating new cytopenias in this population. The successful treatment approach—combining dose-attenuated mini-hyper-CVD with inotuzumab ozogamicin followed by reduced-intensity conditioning allogeneic transplantation with post-transplant cyclophosphamide-based GVHD prophylaxis—demonstrates a feasible strategy for older adults with high-risk secondary B-ALL, achieving durable remission while maintaining acceptable tolerability and warranting further study as a potential treatment paradigm. While lenalidomide maintenance therapy has significantly improved PFS and long-term disease control in patients with MM, becoming a cornerstone of modern myeloma management, prolonged use is increasingly associated with therapy-related secondary malignancies, including rare hematologic neoplasms such as B-ALL. Continued vigilance is essential, particularly in patients with prior exposure to alkylating agents or auto-HCT, where the cumulative risk may be amplified. As overall survival improves, clinicians must balance therapeutic benefits with long-term risks, including rare but serious outcomes such as secondary B-ALL. Patient education, close clinical surveillance, and incorporation of early molecular diagnostics—including cytogenetics, FISH, and NGS—are essential to enable timely detection of hematologic complications. Ultimately, this case contributes to the growing body of literature on lenalidomide-associated secondary malignancies and highlights the importance of individualized survivorship care in the evolving landscape of MM treatment.

## Data Availability

The authors declare that data supporting the findings of this study are available within the article.

## Abbreviations

auto-HCT: autologous hematopoietic cell transplantation
allo-HCT: allogeneic hematopoietic cell transplantation
AML: acute myeloid leukemia
ASCO: American Society of Clinical Oncology
B-ALL: B-cell acute lymphoblastic leukemia
CAR: chimeric antigen receptor
CNS: central nervous system
CSF: cerebrospinal fluid
FDG: fluorodeoxyglucose
FISH: fluorescence *in situ* hybridization
GVHD: graft-versus-host disease
HCT: hematopoietic cell transplantation
HLA: human leukocyte antigen
IgG: immunoglobulin G
MDS: myelodysplastic syndrome
MM: multiple myeloma
MRD: minimal residual disease
NGS: next-generation sequencing
NK: natural killer
PET: positron emission tomography
PFS: progression-free survival
Ph–: Philadelphia chromosome–negative
SPM: second primary malignancy
TdT: terminal deoxynucleotidyl transferase
